# Virulence of *Francisella tularensis* Subspecies *holarctica* Biovar *japonica* and Phenotypic Change during Serial Passages on Artificial Media

**DOI:** 10.3390/microorganisms8121881

**Published:** 2020-11-27

**Authors:** Akitoyo Hotta, Neekun Sharma, Osamu Fujita, Akihiko Uda, Kiyoshi Tanabayashi, Deyu Tian, Akio Yamada, Shigeru Morikawa, Ken Maeda

**Affiliations:** 1 Department of Veterinary Science, National Institute of Infectious Diseases, Toyama 1-23-1, Shinjuku, Tokyo 162-8640, Japan; sharmaneekun@gmail.com (N.S.); esperanz@nih.go.jp (O.F.); auda@niid.go.jp (A.U.); ktana@niid.go.jp (K.T.); tiandeyu2010@126.com (D.T.); s-morikawa@vet.ous.ac.jp (S.M.); kmaeda@niid.go.jp (K.M.); 2 Laboratory of Veterinary Public Health, Graduate School of Agriculture and Life Science, The University of Tokyo, 1-1-1 Yayoi, Bunkyo, Tokyo 113-8657, Japan; vulture-ay@jcom.home.ne.jp; 3 CAS Key Laboratory of Pathogenic Microbiology and Immunology, Institute of Microbiology, Chinese Academy of Sciences, Beijing 100101, China; 4 Department of Microbiology, Faculty of Veterinary Medicine, Okayama University of Science, 1-3 Ikoi-no-oka, Imabari, Ehime 794-8555, Japan

**Keywords:** *Francisella tularensis*, Japan, virulence, phenotypic change, attenuation

## Abstract

*Francisella tularensis* (*F. tularensis*) is the etiological agent of the zoonotic disease tularemia. *F. tularensis* subspecies *holarctica* biovar *japonica* has rarely been isolated in Japan and is considered to have moderate virulence, although the biological properties of fresh isolates have not been analyzed in detail. Here, we analyzed the virulence of two strains of *F. tularensis* subspecies *holarctica* biovar *japonica* (NVF1 and KU-1) and their phenotypic stability during serial passages in Eugon chocolate agar (ECA) and Chamberlain’s chemically defined medium (CDM) based agar (CDMA). C57BL/6 mice intradermally inoculated with 10^1^ colony-forming units of NVF1 or KU-1 died within 9 days, with a median time to death of 7.5 and 7 days, respectively. Both NVF1 and KU-1 strains passaged on ECA 10 times had comparable virulence prior to passaging, whereas strains passaged on ECA 20 times and on CDMA 50 times were attenuated. Attenuated strains had decreased viability in 0.01% H_2_O_2_ and lower intracellular growth rates, suggesting both properties are important for *F. tularensis* virulence. Additionally, passage on ECA of the KU-1 strains altered lipopolysaccharide antigenicity and bacterial susceptibility to β-lactam antibiotics. Our data demonstrate *F. tularensis* strain virulence in Japan and contribute to understanding phenotypic differences between natural and laboratory environments.

## 1. Introduction

*Francisella tularensis* is a facultative intracellular fastidious gram-negative bacterium and is the causative agent of tularemia. Humans are infected with *F. tularensis* through direct contact with infected animals, arthropod bites, contaminated water or food ingestion, or infective aerosol inhalation. The predominant symptoms of tularemia are high fever, lymph node swelling to form abscesses, and ulceration at the site of bacterial entry [1]. Tularemia severity varies depending on the transmission route and bacterial strain involved. In animals, the severity of the disease varies among animal species. In rodents and lagomorphs, severe weakness is followed by a fatal septicemia whereas other animal species are relatively resistant to the infection [2].

*F. tularensis* subspecies (subsp.) *tularensis* and *holarctica* cause human tularemia. *F. tularensis* subsp. *tularensis* is limited to North America, whereas *F. tularensis* subsp. *holarctica* is widely distributed throughout the Northern Hemisphere and Australia [3]. *F. tularensis* subsp. *holarctica* is classified into three biovars, I, II, and *japonica,* based on their biochemistry, erythromycin susceptibility, and the area of isolation [1]. Biovar I is broadly distributed in North America, Russia, Far East Asia, and Europe. Biovar II, which is erythromycin-resistant, has so far been isolated from Eastern Europe, Western Siberia, and Scandinavia. Biovar *japonica* was classically defined as *F. tularensis* distributed in Japan [4]. It is classified as clade B.16 using genome-based classification scheme [5] and some isolates from Australia [3], Turkey [6], and Tibet [7] were genetically classified as biovar *japonica*. Biovar *japonica* is an evolutionary intermediate between subsp. *tularensis* and the other biovars of subsp. *holarctica*. Therefore, it has been proposed as *F. tularensis* subsp. *japonica* [8] although it is not validated. In addition, biovar *japonica* is believed to be less virulent than other biovars due to the relatively mild symptoms of human tularemia found in Japan [9,10].

In Japan, tularemia was first reported as “hare meat poisoning” in 1837 [11]. Approximately 1400 cases of human tularemia have been reported since 1924, however the incidence has become extremely rare since the 1990s [12]. *F. tularensis* was first isolated in 1926 and more than 100 strains have been isolated, mainly during 1950s and 1960s, from humans, hares, ticks, and shrew-moles [13]. These isolates had been maintained by monthly repeated passages on artificial media, such as Eugon agar and pig liver glucose hemoglobin agar, until 1988 [14]. In our previous studies, we found that the Japanese strains differed in their virulence to mice [15], antibiotic susceptibility [16], and variable number of tandem repeat (VNTR) profiles [17]. However, it is not clear whether these differences were altered owing to the bacterial adaptation during passage on artificial media because bacteria can adapt to environments where there is an absence of immune system intervention or stresses are absent. This phenomenon was studied with prototypic strains of *F. tularensis* subsp. *holarctica* and *tularensis*, live vaccine strain (LVS) [18,19] and Schu, respectively [20,21,22], although these strains had been maintained in laboratories for long periods and are probably attenuated [23]. Without understanding the impact of serial passages, results of phenotypic analyses between different laboratories can be confused. In this point of view, phenotypic analyses of wild-type strains and their passaged variants will be helpful to understand the difference between fresh isolates and bacteria that have been maintained in a laboratory for a long period. In 2008 and 2009, *F. tularensis* subsp. *holarctica* biovar *japonica*, NVF1 [24] and KU-1 [25], were isolated from carcasses of Japanese hares and stored in a deep freezer. Thereafter, *F. tularensis* has not been isolated in Japan.

Here we examined wild-type NVF1 and KU-1 to demonstrate their virulence as representative strains of *F. tularensis* subsp. *holarctica* biovar *japonica*. Further, their phenotypic change during serial passages on two different artificial media, Eugon chocolate agar (ECA) and Chamberlain’s chemically defined medium (CDM)-based agar (CDMA), was analyzed. The data are valuable for understanding the virulence of *F. tularensis* in Japan and the phenotypic difference of this bacteria in natural and laboratory environments.

## 2. Materials and Methods

### 2.1. Bacteria Culture

*F. tularensis* NVF1 was isolated in our laboratory from a Japanese hare carcass found in Akita prefecture in 2009 [24]. KU-1, isolated in Aomori prefecture in 2008 [25], was kindly provided by Hisaaki Sato (Kitasato University, Aomori, Japan). The possession and handling of these strains was approved by the ministry of health, labour and welfare in Japan because both are class II specified pathogens under the infectious diseases control law in Japan. *F. tularensis* subsp. *holarctica* biovar II LVS was used as reference for antigenic analysis additionally. It was supplied to Ohara Research laboratory by Rocky Mountain Laboratory, National Institute of Allergy and Infectious Diseases, the United States of America in 1958 and kindly provided by Dr. Hiromi Fujita (Ohara Research Laboratory, Fukushima, Japan). Originally, both NVF1 and KU-1 strains were grown from a single bacterial colony via cultivation on ECA at 37 °C in a biosafety level 3 (BSL3) laboratory at the National Institute of Infectious Diseases (NIID). To obtain passaged variants, numerous bacterial colonies were roughly pooled and cultured on renewed ECA or CDMA continuously. Passage on ECA was performed for 2–4 days, whereas passage on CDMA was performed from 5–7 days. During the cultivation periods, one bacterium grew on ECA and CDMA to approximately 10^8^ colony-forming units (CFU). Every 10 passages, the passaged bacteria were stored at −80 °C in 3% (*w*/*v*) skim milk and 5% (*w*/*v*) sucrose containing distilled water [26]. Passage histories of the variants were designated as EP10 and EP20 if the original strains had been passaged on ECA 10 and 20 times, respectively, and CP50, if the original strains had been passaged on CDMA 50 times. To prepare working stocks of passaged bacteria, bacteria harvested after 48-h cultivation on ECA were suspended in 10% (*v*/*v*) glycerol containing CDM broth at an optical density (OD) at 600 nm of 1.0 and kept at −80 °C until use. For comparison of colony morphology of passaged variants, 5 µL of 10-fold serial diluted bacterial working stock in sterile saline was spotted onto chocolate (II) agar plates (BD, Fukushima, Japan) and incubated at 37 °C for 3–5 days.

### 2.2. Animal Experiments

Mice were used to evaluate the virulence of *F. tularensis* NVF1 and KU-1 at first and then rats were used to evaluate the virulence of NVF1 additionally. Mice were also used for virulence comparison among the passaged variants. Specific-pathogen-free 6-week-old male C57BL/6J mice (body weight 19–23 g), female in-bred Fischer 344 (F344) rats (body weight 109–116 g), and female out-bred Sprague Dawley (SD) rats (body weight 130–138 g) were purchased from Japan SLC, Inc. (Shizuoka, Japan). All the animals were housed in cages with free access to food and water. The inocula was prepared from saline and bacterial working stock described above and the bacterial dose was determined by plating on chocolate (II) agar plates (BD, Fukushima, Japan) at each inoculation. For inoculation of bacteria, the animals were lightly anesthetized with isoflurane. Intradermal (i.d.) injection to mice and rats were performed on their backs. Mice (*n* = 8 per group) were inoculated with 10^0^–10^3^ CFU of NVF1 original or 10^1^ and 10^2^ CFU of KU-1 original per 20 µL. For virulence comparison among passaged variants (*n* = 8 per group), 10^1^ CFU of bacteria per 20 µL were attempted to inoculate. Rats (*n* = 4 to 5 per group) were inoculated with 10^2^ CFU of NVF1 per 20 µL. For intraperitoneal (i.p.) injection, 10^1^–10^4^ CFU per 100 μL of bacteria were administered to F344 rats and 10^3^–10^7^ CFU/100 μL of bacteria were administered to SD rats. Clinical signs and body weights of individual animals were monitored daily. Moribund animals, judged from weight loss exceeding 20% of the body weight of its 0 days post inoculation (dpi), were euthanized by inhalation of isoflurane followed by cervical dislocation and exsanguination. Animals that survived after 21 days post inoculation (dpi) were euthanized. Data for accidental animal death were excluded. The experiments were performed in an animal BSL3 laboratory in strict accordance with the Animal Experimentation Guidelines of NIID and the protocols were approved by the Institutional Animal Care and Use Committee of NIID (Permit Nos. 111116 (11 December 2011), 112065 (5 May 2012), 112137 (1 November 2012), and 116092 (14 July 2016)).

### 2.3. Quantification of Bacterial Burden

Bacterial CFU counts in organs and blood of rats were determined to evaluate the severity of the diseases. Pieces of lung, liver, spleen, and kidney were excised aseptically and their weight measured tissue samples were placed in phosphate-buffered saline (PBS) and mechanically homogenized using a Mini-Beadbeater-1 (BioSpec Products Inc., Bartlesville, OK, USA). The homogenates were serially diluted 10-fold in sterile saline and each dilution was cultured on chocolate (II) agar plates (BD) at 37 °C for 4 days. The colonies on the plates were counted and expressed as CFU per gram of organ or milliliter of blood.

### 2.4. Serological Tests

To confirm infection in animals that survived 21 d, their antibody responses were tested by indirect enzyme-linked immunosorbent assay (ELISA) as described previously [27]. Multi-well plates (Nunc, Roskilde, Denmark) were coated with formalin-killed *F. tularensis* NVF1 whole-cell suspension in 0.1 M sodium bicarbonate solution and incubated overnight at 4 °C. The wells were then washed with PBS supplemented with 0.1% Tween 20 (PBST) and blocked with 3% (*w*/*v*) skim milk in PBST for 1 h at 37 °C. Sera derived from mice and rats were diluted 1:100 in 1% skim milk in PBST and then added to each well and incubated for 1 h at 37 °C. The plates were washed with PBST and incubated with horseradish peroxidase (HRP)-conjugated anti-rat IgG (Santa Cruz Biotechnology Inc., Santa Cruz, CA, USA or anti-mouse IgG (H + L) (Invitrogen, Fredrick, MD, USA) at 1:8000 dilution with 1% skim milk in PBST for 1 h at 37 °C. The reactions were visualized by the addition of diammonium 2,2′-azino-bis(3-ethylbenzothiazoline-6-sulfonate) peroxidase substrate solution (Roche Diagnostics, Mannheim, Germany) and incubated for 30 min at 37 °C; the absorbance at 405 nm was measured by an iMark microplate reader (Bio-Rad, Hercules, CA, USA). Sera of *F. tularensis* immunized mice were used as positive controls and sera from normal mice and rats were used as negative controls. All samples were tested in triplicate, and the samples with OD values over the cutoff value (mean plus 3 standard deviations of negative control) were considered antibody positive.

### 2.5. Determination of Bacterial Growth Curves in CDM Broth

To evaluate growth curves of original and passaged variants of both NVF1 and KU-1 strains, 60 µL of bacterial working stock was inoculated into 3 mL of CDM broth in 16 mL screw cap tubes. The tubes were incubated at 37 °C with shaking at 200 rpm and OD_600_ values were measured every 2 h for 24 h. The test was performed with triplicated samples from at least three independent experiments. Growth curves were obtained using a simple linear interpolation procedure constructed by GraphPad Prism Software ver. 6.0c (La Jolla, CA, USA).

### 2.6. Intracellular Growth Ability of Bacteria in J774.1 Cells

Intracellular growth of NVF1- and KU-1-passaged variants were assessed as reported previously [15]. J774.1 cells (RCB0434; RIKEN Bioresource Center, Ibaraki, Japan) were propagated in RPMI1640 medium (Wako Pure Chemicals; Osaka, Japan) containing 10% (*v*/*v*) heat inactivated fetal bovine serum (FBS) at 37 °C in 5% CO_2_. The cells were grown on 24 well plates (1–2 × 10^5^ cells per well) and were infected with the bacteria at a multiplicity of infection (moi) of 100. This point was designated as time 0. After incubation for 1 h, the cells were washed and fresh 1 mL of 5% FBS-RPMI1640 with gentamicin (10 μg/mL) was added into the wells. The CFU of intracellular bacteria in the J774.1 cells were measured at 2- and 24-h post inoculation (hpi) of bacteria. The cells were washed twice with PBS and were treated with 100 μL of 1% (*w*/*v*) saponin in distilled water for 5 min to lyse the cells [28]. To measure viable bacteria, the lysed cells were serially diluted with saline and 20 μL aliquots of each dilution were spotted onto Chocolate II agar (BD). Bacterial enumeration was performed with triplicated samples at least twice independent experiment. 

### 2.7. Bacterial Viability after Hydrogen Peroxide (H_2_O_2_) Treatment

To assess resistance of the passaged variants to H_2_O_2_, the viable bacterial number following H_2_O_2_ treatment was determined. Approximately 10^7^ CFU of bacteria were kept in PBS (Wako Pure Chemicals) with or without 0.01% (*v*/*v*) H_2_O_2_ (Nacalai Tesque, Inc., Kyoto, Japan) for 4 h at room temperature without shaking, and thereafter, the bacterial CFU in each mixture was determined [29]. 

### 2.8. Sodium Dodecyl Sulfate Poly Acrylamide Gel Electrophoresis (SDS-PAGE) and Western Blotting

To assess the antigenic change on *F. tularensis* during serial passages, whole cell lysate and purified lipopolysaccharides (LPSs) were separated by SDS-PAGE on a pre-cast gel (e-pagel; ATTO Co., Tokyo, Japan) and analyzed by Western blotting [27]. LPSs were purified as described by Barker et al. [30]. PAGE-separated antigens were transferred on a polyvinylidene difluoride (PVDF) membrane (Immobilon: Millipore Corporation, Bedford, MA, USA) and incubated in 3% skim milk PBST at room temperature for 1 h followed by washings with PBST. The PVDF membrane was then incubated with sera obtained from rats survived from the infection with NVF1, in this study and anti-*F. tularensis* monoclonal antibodies (MAbs). Among the six MAbs tested, five MAbs (FB11, M14B11, M11H7, M13A13, and M15C6) recognize LPS whereas the others (M13B10) recognize proteinase K-sensitive components [31]. MAbs M14B11, M11H7, M13A13, M15C6, and M13B10 were derived from hybridoma fused with splenocytes of mice immunized with the reference strain of *F. tularensis* subsp. *holarctica* biovar *japonica* GIEM Miura and myeloma cell line P3X63Ag8.653 (RCB0146; RIKEN Bioresource Center, Ibaraki, Japan). FB11 was purchased from Biodesign International (Saco, ME, USA). After three further washes with PBST, the membranes were incubated with either HRP-conjugated anti-rat IgG (Santa Cruz Biotechnology Inc. Santa Cruz, CA, USA), HRP-conjugated anti-mouse IgG (H + L) (Invitrogen, Fredrick, MD, USA), HRP-conjugated anti-rat IgM (SouthernBiotech, Birmingham, AL, USA) or HRP-conjugated anti-mouse IgM (Santa Cruz Biotechnology) diluted at 1:8000 in 1% skim milk in PBST for 1 h. Finally, MAb-immunoreactive antigens were visualized by incubation with 50 mM Tirs-HCl buffer (pH 7.6) containing 0.02% 3,3′-diaminobenzidine (Wako Pure Chemicals; Osaka, Japan) and 0.003% H_2_O_2_. Precision Plus Protein Prestained Standard (BioRad, Hercules, CA, USA) was used as a molecular weight marker. For further antigenic analyses on LPS, *F. tularensis* LVS was used as reference antigen because it contains free-lipid A molecule [30].

### 2.9. Minimum Inhibitory Concentration (MIC) against Antibiotics

Generally, β-lactam is not recommended for treatment of tularemia whereas some β-lactam susceptible *F. tularensis* were reported [32]. To evaluate the impact of serial passages on antibiotic susceptibility, MICs of the passaged strains against aztreonam, cefotaxime, cefoxitin, cefuroxime, ceftriaxone, and imipenem were determined by use of Etest strips (AB Biomerieux, Solna, Sweden) as reported previously [16]. *Escherichia coli* (ATCC 25922) and *Staphylococcus aureus* (ATCC 29513) were used as control strains.

### 2.10. Variable Number of Tandem Repeat (VNTR) Analysis

VNTR profile is often used for molecular typing of *F. tularensis*. Because our previous study demonstrated high genetic polymorphism among *F. tularensis* in Japan [17], the impact of serial passages on VNTR profile was examined. Genomic DNAs of the passaged variants were extracted using a SepaGene DNA Extraction Kit (Eidia, Tokyo, Japan). Gene fragments encompassing six VNTR loci (Ft-M2, 3, 6, 10, 20, and 25) were amplified using primers specific for sequences flanking each locus [17]. DNA sequencing reactions and analyses were conducted on an ABI Prism 3130 DNA Genetic Analyzer (Applied Biosystems, Foster City, CA, USA) using the ABI Prism BigDye Terminator v3.1 Cycle Sequencing Kit (Applied Biosystems). The number of tandem repeats in each locus was analyzed using CLC Main Workbench 6 software (CLC Bio, Cambridge, MA, USA).

### 2.11. Statistical Analysis

All in vitro experiments were repeated at least three times. Statistical analyses were determined by GraphPad Prism ver. 6.0c Software (La Jolla, CA, USA). Graph data are presented as means ± standard error of the mean (SEM). Multiple *t*-tests were performed to confirm the differences between groups statistically. For comparison of survival in the mice challenge experiments, significance was analyzed by log-rank (Mantel–Cox) test. The median lethal dose (LD_50_) calculation was attempted by the Reed and Muench method [33].

## 3. Results

### 3.1. Virulence of Wild-type F. tularensis Subspecies Holarctica Biovar Japonica

#### 3.1.1. Virulence of NVF1 and KU-1 Strains in Mice

**Figure 1:**
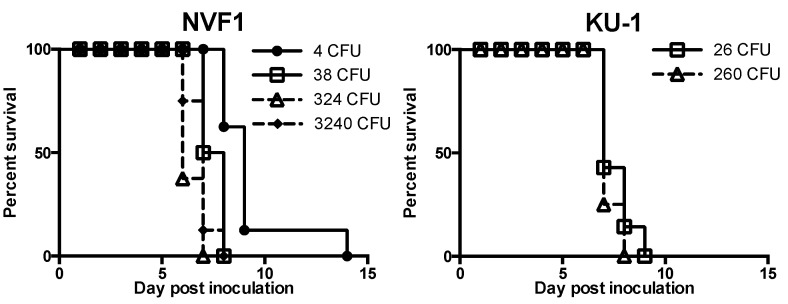
Survival of mice inoculated with *Francisella tularensis* NVF1 and KU-1. C57BL/6J mice (*n* = 7 or 8/group) were challenged with indicated colony-forming units (CFUs) of NVF1 and KU-1 strains intradermally and their survival was monitored over time. The data for NVF1-inoculated mice represent the combined data of two separate experiments.

All C57BL/6 mice inoculated with NVF1 and KU-1 strains by i.d. route exhibited a decrease in body weight (data not shown) and died or became moribund between 6 and 14 dpi. Survival rates of the mice inoculated with indicated doses of the bacteria are shown in Figure 1. The median times to death (MTD) for the mice inoculated with 4, 38, 324, and 3240 CFU of NVF1 were 9, 7.5, 6, and 7 days, respectively, and those of the mice inoculated with 26 and 260 CFU of KU-1 were 7 days for both groups. These results indicate that NVF1 and KU-1 are equally virulent and the LD_50_ of NVF1 by i.d. route to mice was <4 CFU.

#### 3.1.2. Virulence of NVF1 Strain in Rats

**Figure 2:**
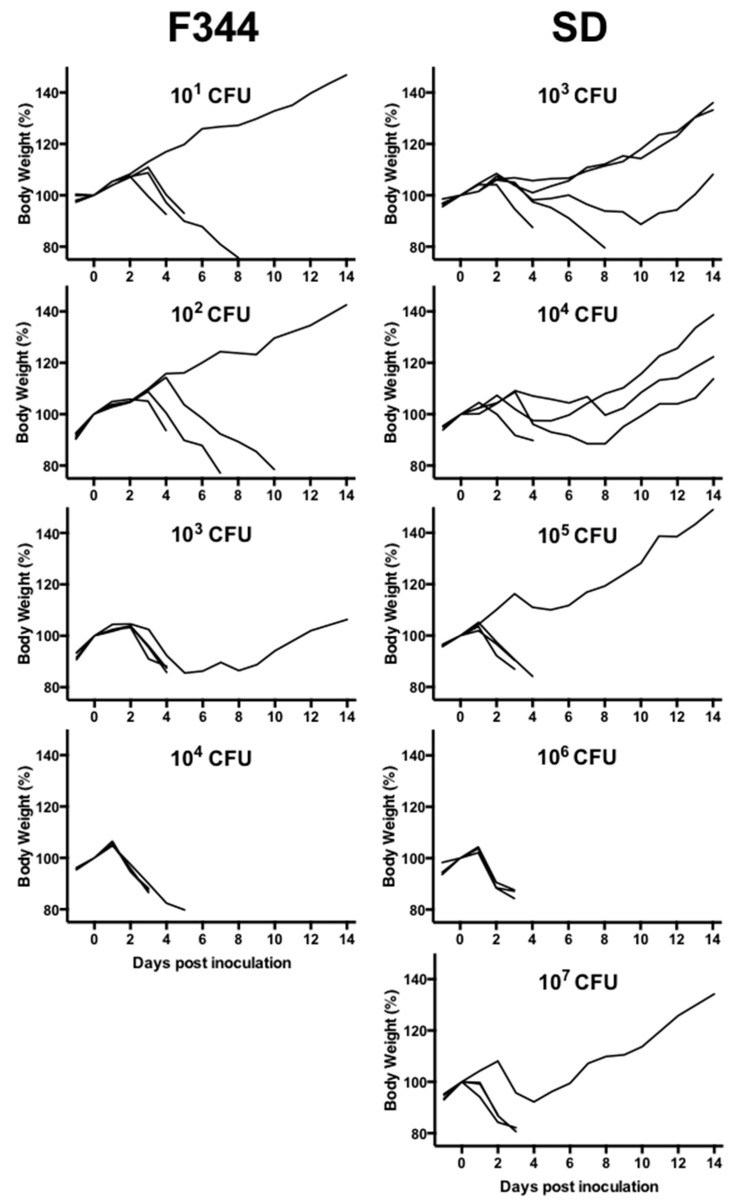
Changes in body weights of rats inoculated with *Francisella tularensis*. F344 and SD rats (*n* = 3–5) were inoculated with NVF1 intraperitoneally. Challenge doses are given in each graph in colony-forming units (CFUs). The graph shows the percent change in body weight relative to day 0 post inoculation for each animal. Data summarized 3 separate experiments.

**Figure 3:**
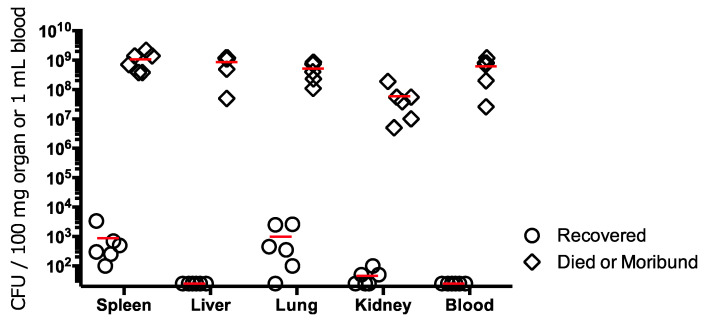
Bacterial burden in organs and blood of recovered and dead or moribund rats. Spleen, liver, lung, kidney, and blood were harvested from SD rats inoculated with 10^3^ and 10^4^ colony-forming units (CFUs) who survived to the end of the experiment period (days post inoculation (dpi: 21) (*n* = 6) and dead or moribund SD rats inoculated with 10^6^ and 10^7^ CFUs (dpi: 2 or 3) (*n* = 6). The mean bacterial burden is shown as a red-colored bar. Bacterial burden in all liver and blood samples of surviving rats were under the detection limit (<50 CFU).

Because results in mice indicated that NVF1 and KU-1 are equally virulent, experimental infection to rats was performed with NVF1 at first. All F344 and SD rats inoculated via i.d. route with 10^2^ CFU of NVF1 lived over 25 days without remarkable symptoms, although seroconversions by ELISA were confirmed. With i.p. inoculation, animals died in all inoculated groups (Table 1 and Figure 2). One of the four F344 surviving rats that maintained body weight after inoculation with 10^1^ CFUs of bacteria showed seroconversion while one of a F344 rat survived from 10^2^ CFU inoculation did not show seroconversion. All F344 rats inoculated with 10^4^ CFUs lost body weight from 2 dpi and died or became moribund between 3 and 5 dpi. In contrast, 10^3^, 10^4^, 10^5^, and 10^7^ CFU inoculated groups of SD rats included one or more survivors. All the surviving rats contained >10^2^ CFUs of bacteria per 100 mg of spleen although bacteria did not grow from liver or blood samples, with <50 CFUs per 100 mg of organs or per milliliter of blood (Figure 3). KU-1 inoculation to rats was not performed due to its unclear dose dependent results compared with the mice experiments.

### 3.2. Phenotype of Variants Passaged on Artificial Media

#### 3.2.1. Colony Morphology and Growth Rate 

**Figure 4:**
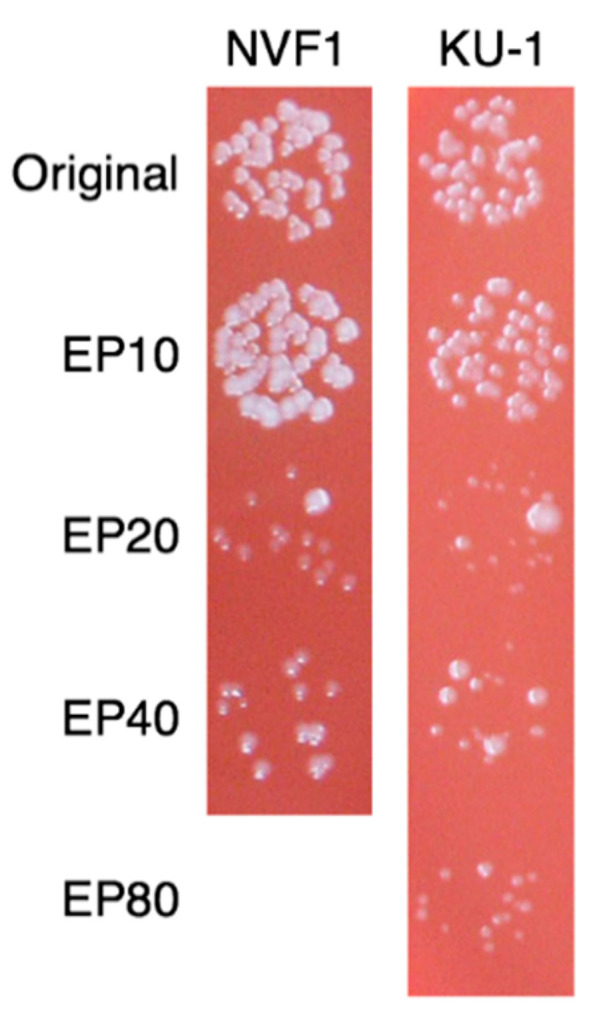
Colony morphology of passaged variants. Five μL of suspended bacteria of original and NVF1 and KU-1 variants passaged on Eugon chocolate agar 10, 20, 40, and 80 times (EP10, EP20, EP40, and EP80 (for KU-1), respectively) were cultured on chocolate agar (II) (BD) for 3 days. Note that original and EP10 form small white convex colonies whereas variants passaged more than 20 times formed tiny flat translucent colonies.

**Figure 5:**
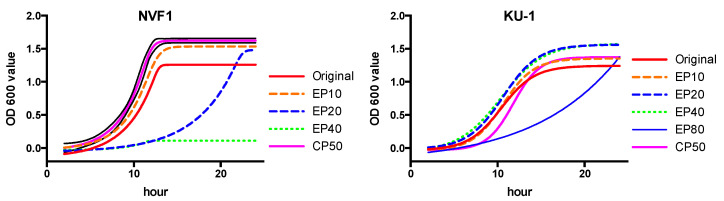
Growth of passaged variants in Chamberlain’s chemically defined medium (CDM) broth. Passaged variants of NVF1 and KU-1 strains were cultured in CDM broth and the optical density (OD) at 600 nm observed for 24 h. Interpolated growth curves were prepared by Graphpad Prism 6.0c. Variants passaged on Eugon chocolate agar 10, 20, 40, and 80 times [EP10, EP20, EP40, and EP80 (for KU-1), respectively] and variant passaged on CDM based agar plate 50 times (CP50) inoculated. NVF1 EP40 failing to grow over 24 h whereas KU-1 variants were attenuated at later passage numbers, with poor growth of EP80 over 24 h.

Following 20 passages on ECA, both NVF1 and KU-1 strains changed their colony size, shape, and color. This morphological change was most apparent after culture on chocolate (II) agar plates (BD). The NVF1 original strain and variant passaged 10 times on ECA (EP10) formed small convex white colonies whereas the variants passaged >20 times (EP20) contained tiny flat translucent colonies (Figure 4). In CDM broth medium, the NVF1 EP20 variant was delayed in growth to the stationary phase and the NVF1 variants passaged 40 times (EP40) failed to grow at all (Figure 5). However, both the KU-1 variants EP20 and EP40 grew in CDM broth medium to the same degree as their original strain, although the variants passaged on Eugon chocolate agar 80 times (EP80) were also delayed in growing to the stationary phase.

#### 3.2.2. Intracellular Growth of the Passaged Variants in J774.1 Cells

**Figure 6:**
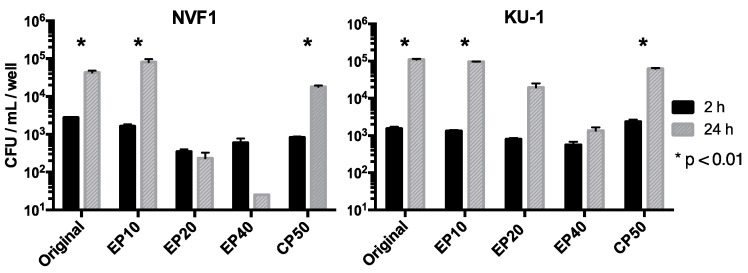
Intracellular growth of passaged variants of NVF1 and KU-1. Original and the variants passaged on Eugon chocolate agar 10, 20, and 40 times (EP10, EP20, and EP40, respectively) and the variants passaged on Chamberlain’s chemically defined medium-based agar 50 times (CP50) of NVF1 and KU-1 strains were inoculated into J774.1 cells. Viable bacteria per well were determined at 2 and 24 h post inoculation. Results are shown as colony-forming unit (CFU)/well. The bars represent the means + SEM of bacterial CFUs from triplicate samples. *, *p*-values less than 0.01 by multiple *t*-test were considered statistically significant. EP20 and EP40 of both NVF1 and KU-1 strains did not significantly increase between 2 and 24 hpi intracellularly.

To assess the virulence of the passaged variants, their intracellular growth abilities in the J774.1 cells were analyzed. CFUs of intracellular bacteria were obtained at 2 and 24 hpi (Figure 6). The EP10 and CP50 variants of both NVF1- and KU-1-passaged strains grew in the J774.1 cells as well as their originals strains. However, variants passaged >20 times on ECA (EP20 and EP40) multiplied poorly in the J774.1 cells, suggesting that the variants passaged on ECA >20 times were relatively less virulent than the originals.

#### 3.2.3. Virulence of the Passaged Variants in Mice

**Figure 7:**
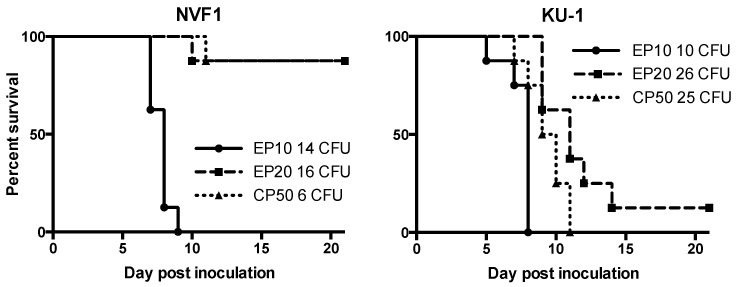
Attenuation of passaged variants of *Francisella tularensis*. Survival rate of mice inoculated with the passaged variants of NVF1 and KU-1. The variants passaged on Eugon chocolate agar 10 times (EP10) and 20 times (EP20), and the variants passaged on Chamberlain’s chemically defined medium-based agar 50 times (CP50) of NVF1 and KU-1 were inoculated intradermally into 6-week-old male C57BL/6J mice (*n* = 8 per group) with indicated colony-forming unit (CFU). The NVF1 EP20 and CP50 variants were significantly attenuated (*p* < 0.001) compared with the NVF1 EP10 variants. Similar results were obtained with the variants of KU-1 EP20 and CP50 (*p* < 0.001 and *p* = 0.008, respectively). *p*-Values less than 0.01 were considered statistically significant.

From the results of intracellular growth analysis, attenuation of EP20 variants of both NVF1 and KU-1 strains were predicted. In addition, we suspected whether CP50 variants for both strains are virulent. Thus, the virulence of the NVF1- and KU-1-passaged variants (EP10, EP20, and CP50) to C57BL/6 mice was compared following i.d. inoculation. All mice inoculated with NVF1 EP10, KU-1 EP10, or KU-1 CP50 died or became moribund within 9 dpi (Figure 7); the MTDs of these inoculated groups were 8, 8, and 9.5, respectively. Statistical analysis indicated that not only EP20 but also CP50 of both strains were significantly attenuated from EP10 variants (*p* < 0.01) and from the original strains (Figure 1 and Figure 7). Surviving mice had lymph node swelling, splenomegaly, or temporal body weight loss suggesting that these mice had been practically infected.

#### 3.2.4. H_2_O_2_ Resistance of the Passaged Variants

**Figure 8:**
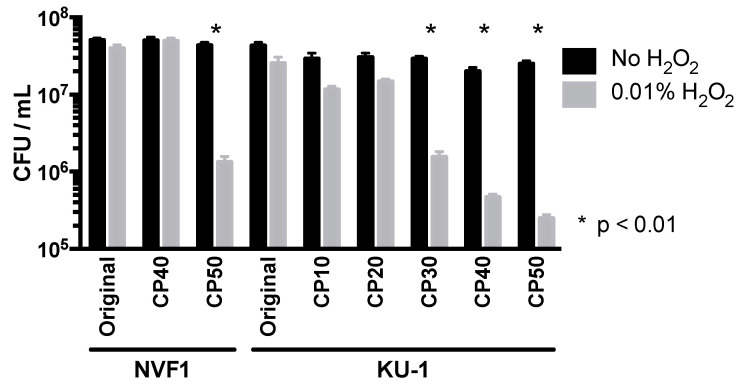
Survival of the passaged variants after exposure to H_2_O_2_. CDM-passaged variants were diluted to approximately 10^7^ colony-forming unit/mL in PBS and thereafter exposed to 0.01% of H_2_O_2_ for 4 h. CFUs are shown as mean + SEM (error bars) based on a minimum of three triplicate observations representing the three independent experiments shown. *, *p*-value less than 0.01 was considered statistically significant. KU-1 variants passaged on Chamberlain’s chemically defined medium-based agar (CDMA) 30 times (CP30), 40 times (CP40), and 50 times (CP50), and NVF1 CP50 were significantly susceptible to H_2_O_2_ while KU-1 variants passaged on CDMA 10 times (CP10), 20 times (CP20), and NVF1 CP40 were not susceptible.

To access the cause of attenuation of CDMA-passaged variants, their H_2_O_2_ resistance was assessed. Both NVF1 CP50 and KU-1 CP50 variants were highly susceptible to H_2_O_2_ and bacterial counts for NVF1 CP50 and KU-1 CP30, CP40, and CP50 decreased approximately 10% following 0.01% H_2_O_2_ treatment whereas the viability of KU-1 passaged on CDMA <20 times and NVF1 strain passaged on CDMA <40 times had not changed (Figure 8). This change of susceptibility to H_2_O_2_ could be one of the causes of attenuation of NVF1 CP50 and KU-1 CP50 to mice.

#### 3.2.5. Antigenic Changes on the Passaged Variants

**Figure 9:**
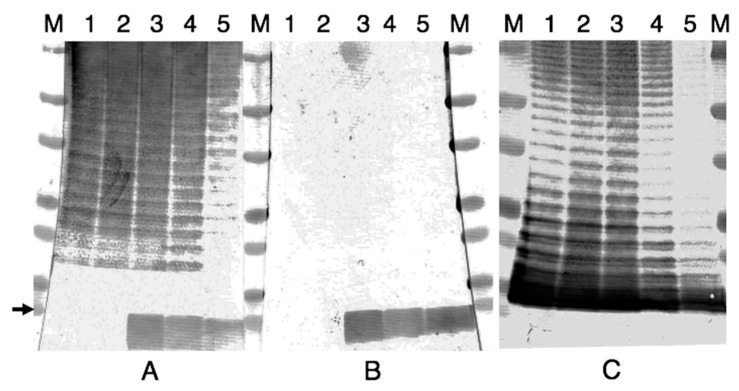
Antigenic change in lipopolysaccharide (LPS) of KU-1 variants passaged on Eugon chocolate agar in Western blots. Purified LPSs of KU-1 original and the variants passaged on Eugon chocolate agar 10, 20, and 80 times (EP10, EP20, and EP80, respectively) and LVS (lanes 1, 2, 3, 4, and 5, respectively) were detected using MAbs M15C6, M13A13, and FB11 [sheets (**A**), (**B**), and (**C**), respectively]. M: molecular weight markers (Bio-Rad) indicate 75, 50, 37, 25, 20, 15, and 10 (arrow) kDa from the top.

Antigenic change was not detected from NVF1 variants passaged on both ECA and CDMA, and KU-1 variants passaged on CDMA from the reaction of MAbs and sera of NVF1 infected rats (data not shown) whereas KU-1 passaged on ECA showed LPS modification. MAbs M15C6 (sheet A) and M13A13 (sheet B) detected a band at approximately 10 kDa of KU-1 EP20 (lane 3) and EP80 (lane 4) whereas this band was not in the KU-1 original (lane 1) or EP10 variant (lane 2) (Figure 9). Similar reaction with LVS, reference antigen contained free-lipid A (lane 5), suggesting these MAbs recognize lipid A molecules. Reactions of MAb FB11 (sheet C) indicate that all antigens contained O-antigen repeat unit structures.

#### 3.2.6. Susceptibility of the Passaged Variants to Antibiotics

Antibiotic susceptibilities of the passaged variants of both NVF1 and KU-1 were compared. Growth of NVF1 EP150, NVF1 CP50, and KU-1 CP50 was not affected by exposure to the β-lactams aztreonam, cefotaxime, cefoxitin, cefuroxime, ceftriaxone, or imipenem; however, KU-1 EP80 became sensitive to those antibiotics. Alteration of MICs was also observed in the KU-1 EP40 variant for cefoxitin, ceftriaxone, cefuroxime, and imipenem but not for aztreonam or cefotaxime (Table 2). These results demonstrate that *F. tularensis* can be susceptible to β-lactams by repeated passages on artificial media.

#### 3.2.7. VNTR Profile

To assess the impact of serial passages to VNTR profile, six VNTR loci of NVF1 EP150, NVF1 CP50, KU-1 EP80, and KU-1 CP50 were amplified and sequenced. The VNTR profiles for Ft-M2, 3, 6, 10, 20, and 25 of NVF1 EP150 and CP50 variants were 8, 3, 4, 5, 11, and 5, respectively, and those of KU-1 EP80 and KU-1 CP50 variants were 8, 3, 4, 16, 11, and 5, respectively. These profiles were identical to the original strain of NVF1 and KU-1 strains [34]. These six VNTR loci would be relatively stable during serial passages on artificial media.

## 4. Discussion

In Japan, a number of *F. tularensis* strains had been isolated since 1926 and had been maintained by monthly repeated passages on artificial media until 1988. Most of the original isolates had undergone phenotypic change because of the high repeated passage [14]. Thus, actual virulence of *F. tularensis* subsp. *holarctica* biovar *japonica* is not understood. This study focused on confirmation of virulence of the two wild-type *F. tularensis* subsp. *holarctica* biovar *japonica*, NVF1 and KU-1 strains and their phenotypic changes during serial passages on artificial media. 

All C57BL/6 mice inoculated with the original NVF1 and KU-1 via i.d. injection had succumbed to infection within 14 dpi (Figure 1). This indicates that <10 CFU of NVF1 or KU-1 are sufficient to infect mice by all routes because bacteria doses required to induce tularemia by i.d. injection are higher than those by other routes [35]. The MTDs for the mice that received 10^1^ CFU of NVF1 or KU-1 (7.5 and 7, respectively) were comparable with those inoculated with other subsp. *holarctica* strains including a Swedish strain (FSC108) [36] and two US strains (KY99-3387 and MI00-1730) [37]; the MTDs mice infected with these strains were between 7 and 9, whereas those of the mice infected with subsp. *tularensis* were between 5.5 and 6.4 [37]. Therefore, NVF1 and KU-1 are likely as virulent as *F. tularensis* subsp. *holarctica* isolated in Europe and North America.

In the experimental rat infection model, NVF1 did not cause lethal infection by i.d. inoculation, although lethal infection was obtained with i.p. inoculation (Table 1 and Figure 2). SD rats were more resistant to infection from i.p. injection compared with F344 rats [38] where all F344 rats inoculated with 10^1^ CFU of *F. tularensis* subsp. *holarctica* (FSC108) by i.p. succumbed within 10 dpi whereas SD rats survived following a 10^5^ CFU inoculation. Similar to their report, our results showed that the spleen and lungs were favorable organs for *F. tularensis* to persist in recovered rats (Figure 3). Although the lethal rates in rats obtained in this study were not dose dependent, Kreizinger et al. reported similar results with Italian (subsp. *holarctica* biovar I) and Hungarian strains (subsp. *holarctica* biovar II) [39]. Thus, compared with i.d. inoculation to mice, i.p. inoculation to rats is not an appropriate method for virulence comparison of *F. tularensis* subsp. *holarctica*. Reproductive hormones may regulate the activity of intraperitoneal macrophages and growth of *F. tularensis* as mentioned by Conlan et al. [40]. It would be interesting to understand the susceptible difference by analysis of regional immune responses to *F. tularensis* in mice and rats. 

Passaged variants of *F. tularensis* subsp. *holarctica* biovar *japonica* in this study underwent changes in various properties. Changes of colony morphology (Figure 4) and growth rate in CDM (Figure 5) suggest that some bacterial populations can alter their regulation of metabolic genes [41]. The decreased growth rate of passaged variants in J774.1 cells (Figure 6) indicates that both NVF1 and KU-1 strains had attenuated between 11 and 20 passages on ECA. However, the intracellular growth ability (Figure 6) and virulence in mice (Figure 7) of CP50 variants for both NVF1 and KU-1 suggest that intracellular growth ability does not entirely reflect virulence. Susceptibility to H_2_O_2_, reactive oxygen species, may be one of the causes for attenuation of CP50 variants in mice. Thus, both intracellular growth ability and resistance against reactive oxygen species would be essential indicators in accessing the virulence of *F. tularensis*; on the basis of the susceptibility to H_2_O_2_, KU-1 CP30 and CP40 are likely to be attenuated as well as KU-1 CP50 (Figure 8).

Changes of antigenic property and antibiotic susceptibility were limited in KU-1 variants passaged on ECA. MAb immunogenicity of M13A13 and M15C6 in Western blots (Figure 9) indicates that the LPS of KU-1 EP20 and EP80 differs from those of the original KU-1 and EP10 variants whereas these were similar with those of LVS antigenically. This phenomenon would be different from phase variation reported by Cowley et al. [18] because reactions of MAb FB11 suggested all antigens contained LPS O-antigen repeat units similarly. Other LPS modifications in *F. tularensis* subsp. *holarctica* has been studied with wild-type strain (1547-57) [42] and attenuated strain, LVS [43]. The wild-type strain contains four long chain fatty acids (16 and 18 carbons), lacks a 4′-phosphate residue, and is partially substituted with phosphate or phosphogalactosamine at the 1-position of the diglucosamine backbone [42] whereas LVS contained one or more fatty acids of a shorter length as well as a free reducing terminus with no phosphate or sugar substitutions [43], so called free lipid A. This structural difference was reportedly due to strain variation or different lipid A isolation and purification steps [42], rather than the analysis method [44]. In our preliminary experiment, MAbs M13A13 and M15C6 strongly reacted with pellets of LPS boiled in 6% acetic acid, lipid A [45], whereas other MAbs did not. Thus, our findings suggest that the structural differences in lipid A is due to excess passages of bacteria on artificial media. Further chemical analyses on fatty acid and sugar substitutes, and regulatory analyses on genes involved in lipid A biosynthesis, such as, *lpxL1*, *lpxL2*, *kdtA* [30], *kdhAB* [46], *lpxC*, *lpxF* [47], and *flmK* genes [48], would be of interest. Thus, attenuated *F. tularensis* could be partly selected by the immunogenicity of MAbs M13A13 and M15C6. Because lipid A is the hydrophobic anchor of LPS that makes up the outer monolayer of the outer membranes [49], structural change on lipid A may influence the bacterial outer membrane permeability and its susceptibility to β-lactams antibiotic as observed in this study (Table 2). Beta-lactamases of *F. tularensis*, FTU-1 [50] and Bla2 [51], would not be related to the MIC changes because these enzymes are ineffective against most third-generation cephalosporins, such as cefotaxime and ceftriaxone.

Our results demonstrate that the mechanisms for phenotypic change in *F. tularensis* differ among strains and media used for passages. In general, the switching event in bacterial phenotypic change per cell is a stochastic event, and its frequency can be modified by specific growth conditions or physiological states [20]. Therefore, both genetic and epigenetic studies on passaged variants are necessary to clarify the mechanisms of the phenotypic change. Complete genome sequences of both NVF1 and KU-1 strains have recently been released [52], and further study will be targeted to genetic mutations on variants that may be detected from variants passaged 30 times as reported [53]. On the other hand, the VNTR profile was barely affected by extensive passages on artificial media as previously reported [54,55]. However, most Japanese strains were suspected to be passaged over 300 times [14]. Further analyses may be necessary to confirm the high genetic polymorphism among *F. tularensis* in Japan.

## 5. Conclusions

Compared with other reports, we found that *F. tularensis* subsp. *holarctica* biovar *japonica* were not specifically less virulent to mice and rats than other biovars of subsp. *holarctica*. Epidemiological differences of tularemia between Japan and European countries are therefore possibly not due to the virulence of distributed *F. tularensis* strains. Other factors such as population densities of the pathogen, reservoirs, and vectors; life style and sensitivity of people; and bacterial adaption to environmental factors may cause the epidemiological differences, although direct comparative study with the bacteria passaged <10 times is necessary. Our data are valuable for comparing the phenotype of *F. tularensis* in natural and laboratory environments as laboratory strains may be attenuated with alterations to growth ability, antigenicity, and susceptibility to antibiotics. In this context, NVF1 and KU-1 strains will be used as a standard for wild-type *F. tularensis* subsp. *holarctica* biovar *japonica*.

## Figures and Tables

**Table 1:** Virulence of *Francisella tularensis* NVF1 to rats.

Route	Rat Strain	Challenge Dose (Colony-Forming Unit)	No. of Survivors/Total	Day of Death (Day Post Inoculation)	Initial Day of Body Weight Loss (Day Post Inoculation)
i.d.	F344	3.2 × 10^2^	4/4	−	−
	SD	3.2 × 10^2^	4/4	−	−
i.p.	F344	3.8 × 10^1^	1/4	4, 5, 8	3, 4, 4
		1.1 × 10^2^	1/4	4, 7, 10	4, 4, 5
		1.1 × 10^3^	1/4	4, 4, 4	3, 3, 3, 4
		3.8 × 10^4^	0/4	3, 3, 3, 5	2, 2, 2, 2
	SD	1.4 × 10^3^	3/5	4, 8	3, 4, 4, 4
		1.4 × 10^4^	3/4	4	2, 3, 4, 8
		1.4 × 10^5^	1/4	3, 4, 4	2, 2, 2, 4
		1.1 × 10^6^	0/3	3, 3, 3	2, 2, 2
		1.1 × 10^7^	1/4	2, 3, 3	1, 2, 2, 3

Data summarized 4 separate experiments. −: Not observed.

**Table 2:** Minimum inhibitory concentration (MIC) (μg/mL) of *Francisella tularensis* KU-1 passaged variants.

Antibiotic	MIC of KU-1 *					MIC Range Among Japanese Strains [16]
	Original	EP20	EP40	EP80	CP50	
Aztreonam	≥256	≥256	≥256	**8**	≥256	0.75 to ≥256
Cefotaxime	≥256	≥256	≥256	**0.38**	≥256	0.047 to ≥256
Cefoxitin	≥256	≥256	**2**	**2**	≥256	0.25 to ≥256
Ceftriaxone	≥32	≥32	**0.25**	**0.25**	≥32	0.047 to ≥32
Cefuroxime	≥256	≥256	**12**	**12**	≥256	0.5 to ≥256
Imipenem	≥32	≥32	**0.5**	**0.5**	≥32	0.047 to ≥32

* MICs of KU-1 original and KU-1 variants passaged on Eugon chocolate agar 20, 40, and 80 times and the variants passaged on Chamberlain’s chemically defined medium-based agar 50 times (EP20, EP40, EP80, and CP50, respectively), where Bold indicates the MIC different from that of original.
